# Does Elemental Sulfur Act as an Effective Measure to Control the Seasonal Growth Dynamics of Potato Tubers (*Solanum tuberosum* L.)?

**DOI:** 10.3390/plants11030248

**Published:** 2022-01-18

**Authors:** Witold Grzebisz, Karolina Frąckowiak, Tomasz Spiżewski, Katarzyna Przygocka-Cyna

**Affiliations:** 1 Department of Agricultural Chemistry and Environmental Biogeochemistry, Poznan University of Life Sciences, Wojska Polskiego 28, 60-637 Poznan, Poland; katarzyna.przygocka-cyna@up.poznan.pl; 2 Yara International ASA Drammensveien, 131 0277 Oslo, Norway; karolina.frackowiak@yara.com; 3 Department of Vegetables Production, Poznan University of Life Sciences, Wojska Polskiego 28, 60-637 Poznań, Poland; tomasz.spizewski@up.poznan.pl

**Keywords:** growth dynamics, dry matter partitioning, tuber vs. non-storage organs competition, nitrogen rates, elemental sulfur

## Abstract

The in-season dynamics of potato tuber biomass (TTB) growth requires effective nitrogen (N) control. This hypothesis was tested in 2006 and 2007. The two-factorial experiment with two rates of N (60, 120 kg ha^−1^) and sulfur (S; 0, 50 kg ha^−1^) was carried out in the split-plot design. The third factor was the sampling of plants at 10-day intervals. The collected plant material was divided into leaves, stems, stolons + roots, and tubers. The seasonal trend of TTB was linear, while the biomass of leaves, stems, and stolons + roots was consistent with polynomial regression models. TTB was controlled by (i) the date of potato growth after emergence, when the TTB exceeded the leaf biomass (DAE_crit_); (ii) the stem growth rate; and iii) the share of stems in the total potato biomass. TTB growth was reduced when DAE_crit_ preceded the DAE_op_ for leaf biomass, determining its maximum. This phenomenon appeared in 2007 on plots fertilized only with N. The absolute growth rate of the stem biomass, exceeding ¼ of that of the tuber biomass in the descending phase, resulted in an increased and prolonged share of stems in the total potato biomass, which ultimately led to a decrease in tuber yield. The use of sulfur to balance the N, applied effectively, controlled the growth rate of potato organs competing with tubers.

## 1. Introduction

Plant growth depends on the net assimilate production by its photosynthetic tissues, and on their subsequent partitioning between other assimilate-dependent and actively growing tissues [1,2]. The capacity of plant tissues to produce the assimilates is expressed as its source strength, which is a result of the size of the source and its activity. Sink tissues consume the assimilates provided by the plant source mainly for growth. The sink capacity of plant tissues to reuse assimilates is defined as the sink strength, which is determined by the size of the sink and its activity [2].

There is no doubt that potato tubers are a net sink and mature leaves are the pure source organ [3]. The growth continuity of potato organs undergoes change as a result of the transformation of the underground stems, i.e., stolons into tubers. This process, driven by plant hormones, is, however, sensitive to external factors like temperature, soil moisture, and the supply of nutrients [4]. The availability of N to the potato does not affect the number of leaves but increases their surface area [5]. Deficiency in two key growth factors, i.e., water and N, is the main reason for source strength disturbance, which subsequently has a negative effect on the production of assimilates. The in-season variability in the quantity of produced assimilates impacts the structure of their subsequent partitioning between plant tissues [6].

An efficient strategy to increase potato yield, as suggested by Katoh et al. [7], consists of two steps. Firstly, the growth processes taking place in the period extending from the early growth stage to the tuber-bulking stage should lead to the intensification of assimilate production in the source tissues. The intensification of the source activity can be achieved as a result of its higher net photosynthetic activity by increasing the leaf surface area. Secondly, the growth processes should be oriented towards increasing the size of the tubers in the early bulking phase. This concept has, however, some weak points. The key target can only be achieved as long as there is an adequate and balanced supply of N [8,9,10]. The targets of other nutrient applications are to increase both N-use efficiency (NUE) and the rate of assimilates transported to the growing tubers [11,12]. Enhancement of the source activity based only on the N dose may disturb the processes responsible for tuber initiation [4].

Engels and Marschner [13] stated that the final tuber yield depends on the size and growth rate of young tubers. The potential of the juvenile potato tuber for assimilate absorption depends, however, on its N content [14]. The realization of the tuber production potential depends first of all on the supply of potassium (K) [11,15]. Synchrony between the photosynthetic activity of leaves and tuber growth requires at least a good supply of other nutrients, such as K, magnesium (Mg), and phosphorus (P) [1,11,16]. K and Mg are responsible for the transport of assimilates in the phloem from the leaves to the enlarging tubers [16,17]. K shortage leads to a tremendous loss of yield, irrespective of the primary N status in young tubers [14].

The N supply to the potato during the growing season can be effectively controlled by a sound use of other agronomic measures. The basic one is regulation of the N dose. It is well-documented that too low an N supply to potatoes in the period of its early growth may result in a reduction of the photosynthetic area of the plants. On the other hand, too high an N rate may disturb plant growth and the processes of potato tuberization [4,18,19]. Another agronomic solution focused on the effective control of the N supply is the use of elementary S (S^0^). It has been well-documented that S^0^ oxidation is mediated by soil bacteria. The end products are a sulfuric ion (SO_4_^2−^) and H^+^ [20]. Potatoes require a good supply of S for effective growth and to lower the soil pH, especially in soils with a high pH. In conditions of good S supply, a simultaneous increase in the efficiency of N can also be expected [21,22].

White et al. [6] concluded in an extensive review that crop productivity is driven by growth limitations imposed by the degree of source and sink balance during development. The potato tuber is the pure sink tissue of this crop. All other tissues begin their life cycle as sink organs [7]. Thus, the following key question arises: “Is the tuber sink strength limited by the strength of other potato sinks or vice versa?” Further minor questions are as follows:

1. Does the pre-tuberization period of potato growth impact the development of tuber sink strength?
2. To what extent and for how long do the temporary sinks limit the tuber sink strength?
3. Is it possible to effectively control the sink strength of potato tissues competing with the tubers by agronomic measures?

The objective of the study was to (i) quantify the seasonal growth trends in the biomass of potato organs competing with tubers and (ii) evaluate the impact of elemental sulfur on the in-season relationships between the biomass of potato organs.

## 2. Results

### 2.1. In-Season Trends of Tuber Yield Development

**Figure 1:**
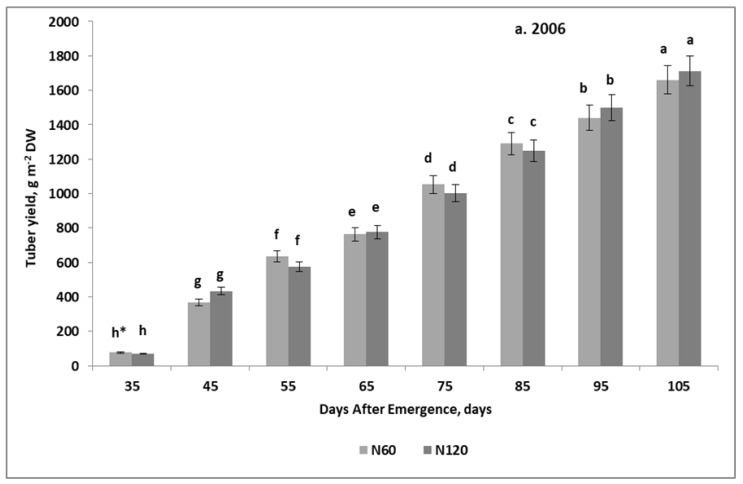

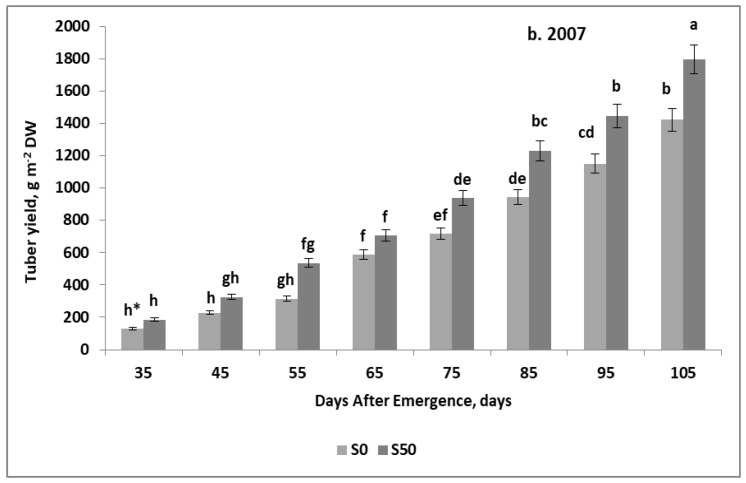
In-season development of the temporary tuber biomass. (**a**) 2006; (**b**) 2007. * HSD—calculated separately for each year; letters indicate significant differences between treatments (*p* < 0.05). Legend: N60, N120—nitrogen rates of 60 and 120 kg ha^−1^; S0, S50—sulfur rates of 0 and 50 kg ha^−1^. Vertical bars represent standard error.

The distinctly different annual growth patterns of TTB response to experimental factors were the basis for the analysis of tuber yield growth trends for each study year separately. In 2006, TTB responded significantly to the N × T interaction, and indeed to T (sampling interval (Table 1, Figure 1a). The general trend of potato tuber yield development during the growing season was as follows:

(1) TTB = 22.49 DAE − 661.2 for R^2^ = 0.99, *n* = 8, and *p* ≤ 0.01

In the 2007 growing season, the increase in TTB depended on the effect of the S × T interaction (Table 1, Figure 1b). A significant and simultaneously progressive increase in TTB was recorded from the 35th DAE. But the variation due to S treatments was revealed from DAE 85. The effect of S assessed only against the background of N fertilization, i.e., for the S0 plot, increased from 56 g m^−2^ DW in the 35th DAE to 372 g m^−2^ DW at harvest. The observed differences resulted from a significantly different rate of TTB increase in the growing season:

(2) S0: TTB = 18.65 DAE − 618.9, for R^2^ = 0.98, *n* = 8, and *p* ≤ 0.01

(3) S50: TTB = 22.84DAE − 703, for R^2^ = 0.99, *n* = 8, and *p* ≤ 0.01

The growth rate of TTB in the S0 treatment was 18% lower compared to that with added S. The value of the direction coefficient (CD) of the equation developed for the S50 was almost the same as in 2006.

### 2.2. In-Season Trends in Potato Non-Storage Organs Development

**Figure 2:**
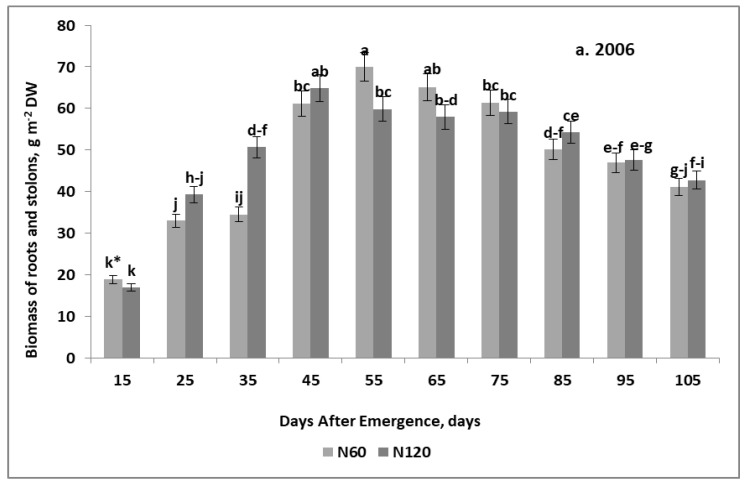

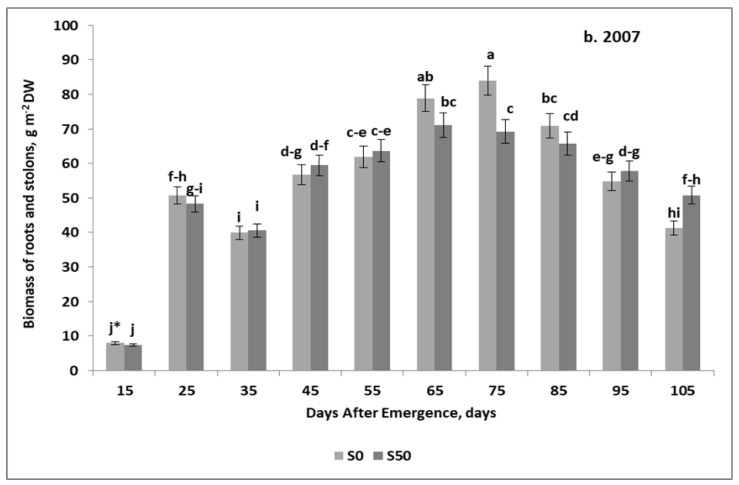
Seasonal trends of roots and stolons biomass growth. (**a**) 2006; (**b**) 2007. * HSD—calculated separately for each year; letters indicate significant differences between treatments (*p* < 0.05). Legend: S0, S50—sulfur rates of 0 and 50 kg ha^−1^. Vertical bars represent standard error.

Seasonal trends in the growth of the non-storage potato organs are presented only for treatments decisive for the temporary tuber yield. In 2006, the whole period of roots + stolons biomass (RS) growth can be divided into three sub-phases (Figure 2a):

(1) Progressive, which lasted until the 45th DAE;
(2) Stagnation, which lasted from the 45th to the 75th DAE;
(3) Depressive, which started from the 75th DAE and lasted until the potato harvest.

**Figure A1:**
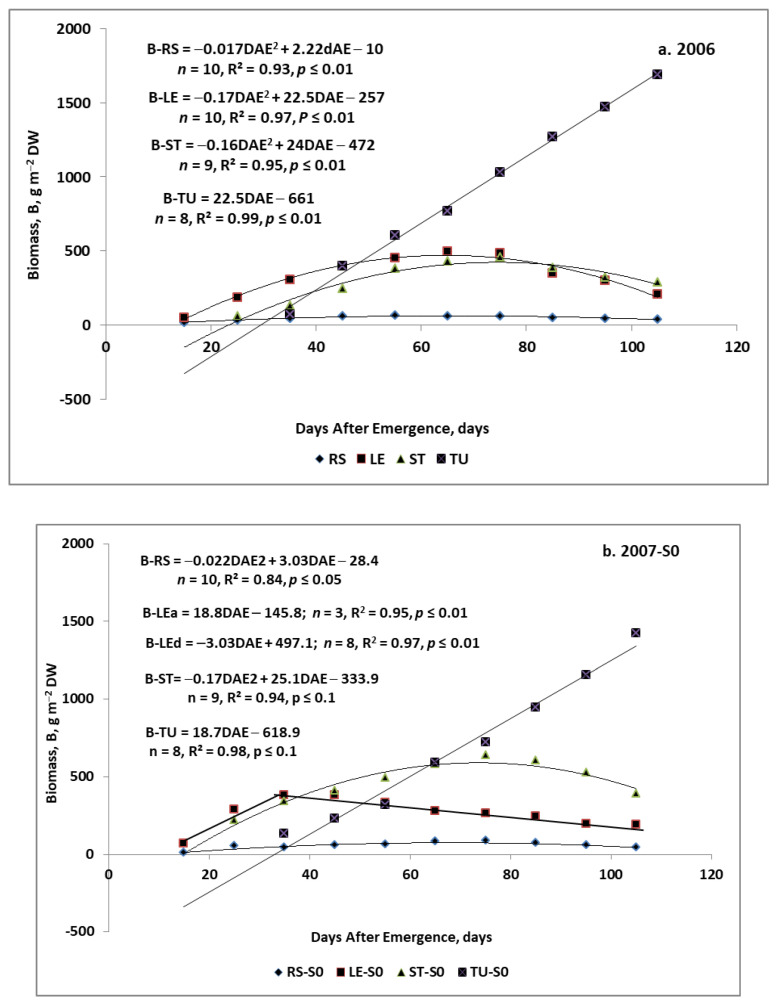

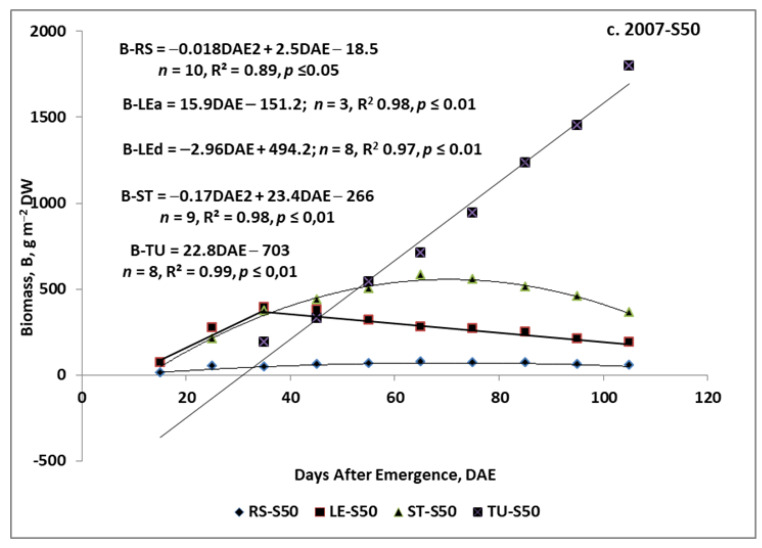
Trends in the biomass of potato organs increase during the growing season. (**a**) 2006; (**b**) 2007-S0; (**c**) 2007-S50. Legend: RS—roots and stolons, LE—leaves, ST—stems, TU—tubers; S0, 50—sulfur rates of 0, 50 kg ha^−1^; B-STa—ascending phase; B-STd—descending phase.

The 2006 growth trend of RS was best described by the quadratic regression model (see Figure A1a for details). The maximum RS of 62.5 g m^−2^ was recorded on the 66th DAE. In 2007, the seasonal growth course of these organs changed significantly on the 35th DAE (Figure 2b). In this particular phase, there was a significant decrease in RS biomass compared to its value observed on the 25th DAE. In subsequent stages, the increase in RS was consistent with the quadratic regression model. The regression models presented below do not take into account the data from the 25 DAE:

(4) S0: B-RS = −0.024DAE^2^ + 3.35DAE − 41.9 for *n* 9, R^2^ = 0.93, *p* ≤ 0.01

(5) S50: B-RS = −0.019DAE^2^ + 2.79DAE − 30.4 for *n* 9, R^2^ = 0.99, *p* ≤ 0.01

In the ascending phase of RS growth, its main characteristic feature was a much steeper increase in biomass in the S0 variant in relation to the S50 variant. A reverse trend was observed in the descending phase of RS growth. In the case of the S0, the RS maximum of 75 g m^−2^ DW was reached on the 70th DAE. For the S50, the net increase in RS of 72 g m^−2^ was recorded on the 73rd DAE.

**Figure 3:**
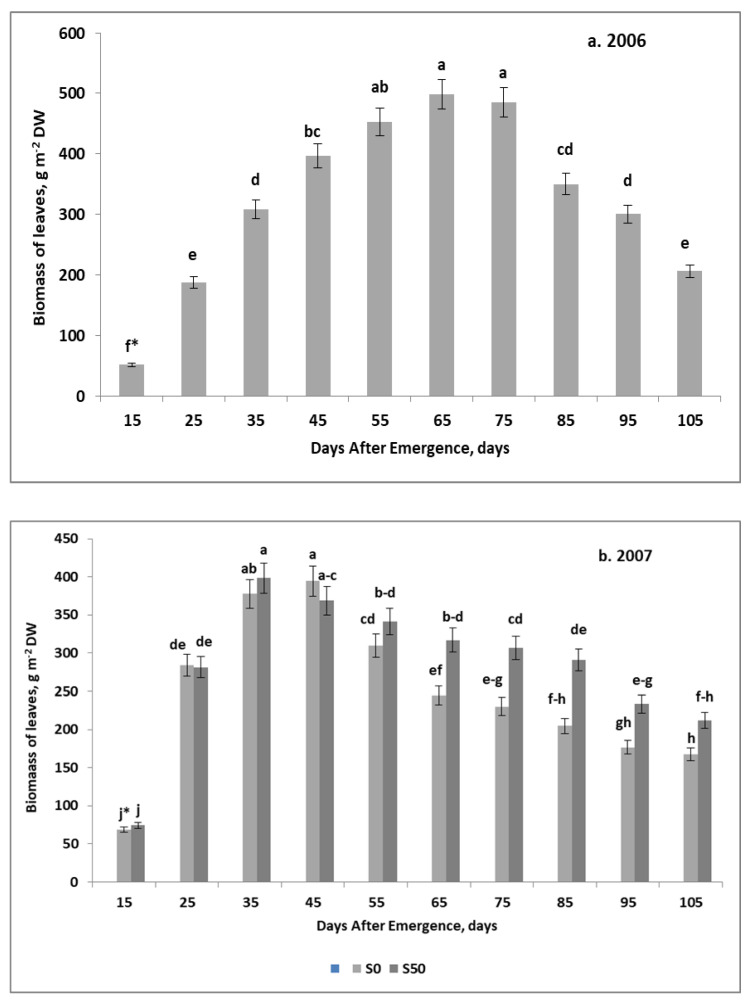
Seasonal trends of leaves biomass growth. (**a**) 2006; (**b**) 2007. * HSD—calculated separately for each year; letters indicate significant differences between treatments (*p* < 0.05). Legend: S0, S50—sulfur rates of 0 and 50 kg ha^−1^. Vertical bars represent standard error.

Seasonal growth trends of potato leaf biomass (LE) were completely different in the studied years. In 2006, the increase in LE during the growing season was best reflected by the quadratic regression model (Figure 3a). The progressive increase in LE continued up to the 65th DAE, reaching a maximum of 470 g m^−2^ (see Figure A1a for details). In 2007, the seasonal course of LE was best represented by the double-linear model (Figure 3b, and see Figure A1b,c for details). The most important attributes of the models obtained, regardless of the fertilization treatments, are:

(1) Steep initial rise, peaking just after the 35th DAE;
(2) A slight decline, after reaching the maximum biomass;
(3) Significantly less decline on the S50 variant compared to the S0.

The ascending sub-phase of this model was short and steep. The maximum LE for both treatments was recorded on the 30th and 34th DAE and amounted to 407.7 and 393.3 g m^−2^ DW for the S0 and the S50, respectively. In contrast, the descending sub-phase of LE growth was long and smooth. A slightly slower rate in LE decline was recorded for the S50 in relation to the S0. During this period, a distinct and much higher biomass was observed in the S50. The observed difference was most pronounced between the 65th DAE and the 85th DAE.

**Figure 4:**
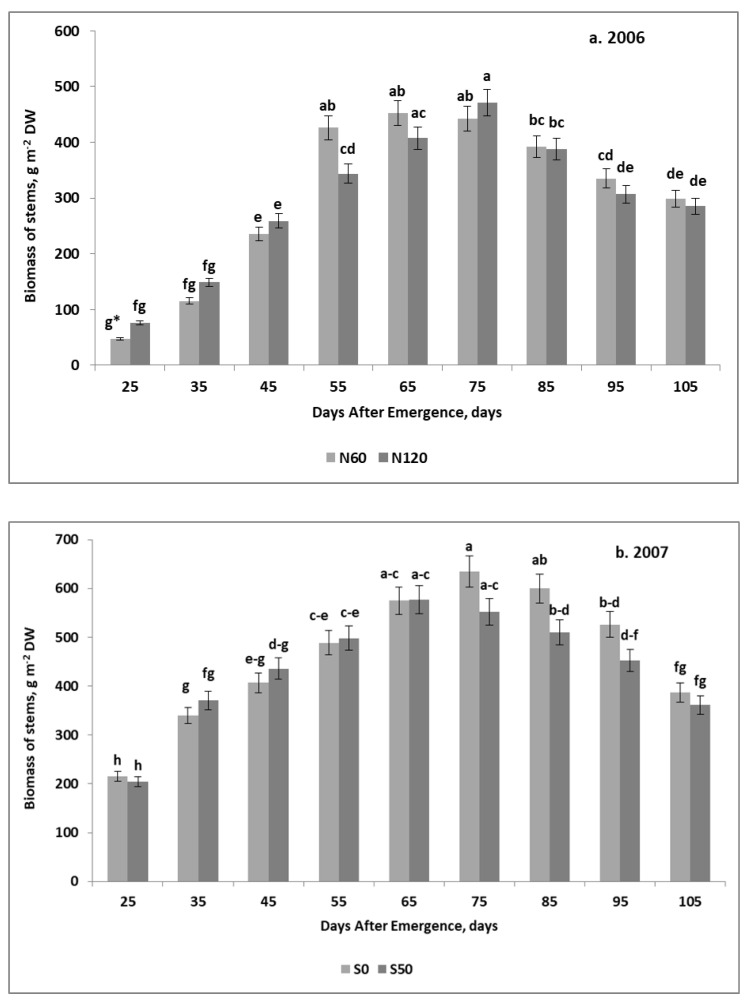
Seasonal trends of stems biomass growth. (**a**) 2006; (**b**) 2007. * HSD—calculated separately for each year; letters indicate significant differences between treatments (*p* < 0.05). Legend: S0, S50—sulfur rates of 0 and 50 kg ha^−1^. Vertical bars represent standard error.

Seasonal growth trends of stems biomass (ST), regardless of the year and applied fertilizers, were consistent with the quadratic regression model. In 2006, a maximum ST of 425.2 g m^−2^ DW was recorded on the 75th DAE (Figure 4a). In 2007, the course of the ST was significantly influenced by fertilization. The differences between the sulfur treatments up to the 75th DAE were slight or negligible (Figure 4b). Significant differences resulting from S application were noted during in the descending phase of the stem biomass growth. These differences are reflected in the biomass maxima, and respective DAE optima. In the case of S0, the ST maximum of 590.4 g m^−2^ DW was reached in the 74th DAE. The corresponding values for the S50 were 555.1 g m^−2^ DW and DAE 76.

### 2.3. Competition Analysis of Tubers and Non-Storage Potato Organs Growth

The seasonal pattern of TTB growth, in spite of being significantly influenced by weather, was consistent with the linear regression model (Figure 1).

The source–sink relationship between enlarging tubers and competitive organs was analyzed in two distinct ways. The first criterion of the studied competition between potato parts focused on the determination of the critical day of potato vegetation i.e., the DAE_crit_ value. DAE_crit_ defines the potato vegetation period, in which the tuber biomass exceeded that of the competing part of the potato. The presented results refer to treatments, which were found to be decisive for the yields of tubers (Figure 1).

Diagrams of the growth of particular potato organs treated as a function of time (T, DAE) are presented in Figure A1a–c. The models obtained in 2006, as shown earlier in Section 2.2, are best described by the quadratic regression model. DAE_crit_ was the longest for LE, 10 days shorter for stems and 14 days shorter for roots and stolons (Table 2). DAE_op_, indicating a period of net growth of potato organs competing with tubers, appeared for all potato organs at almost the same time, i.e., around the 66th DAE. The values of the next index, i.e., DAE_diff_ were the longest for RS and the shortest for LE. In 2007, the DAE_crit_ indices showed clearly different trends compared to 2006. First, the DAE_crit_ values for LE appeared much earlier than in 2006. Secondly, they appeared, regardless of S fertilization, after the leaves reached the maximum biomass, i.e., DAE_op_. As a consequence, DAE_diff_ showed a negative value, especially in the S0 treatment, which preceded the DAE_op_ by 10 days. In the case of the S50, the DAE_diff_ was also negative, but it was approaching zero. It should be emphasized that both indices, i.e., DAE_op_, and especially DAE_crit_ for S × T interaction, appeared much later than those observed in 2006. The DAE_crit_ for the S0 was longer by three, and for the S50 by two weeks. In the case of DAE_op_, these differences were 7.3 and 4.1 days, respectively.

The second criterion for assessing internal competition between the growing organs of the potato was the analysis of the source–sink relationship. In the performed allometric analysis, the potato tuber was treated as a pure sink of assimilates, and the potato’s non-storage organs as their source for the growing tubers. The basic parameters of the solved regression models were (i) optimal, i.e., temporary tuber biomass (TTB_crit_), and (ii) the corresponding, maximum biomass of the competing potato part (B_max_). The intersection of these two regression equations determined the TTB_crit_ value. The slope of the obtained linear models was used as the competition index (CI) between the tuber growth rate and the growth rate of the remaining potato organs (Table 3). In 2006, the values of the CI indices for potato organs competing with tubers were as follows:

1. Ascending phase: 0.04 (RS) < 0.27 (LE) < 0.45 (ST, g g^−1^ tuber DW);
2. Descending phase: −0.41 (LE) < −0.26 (ST) < −0.03 (RS g g^−1^ tuber DW).

**Figure 5:**
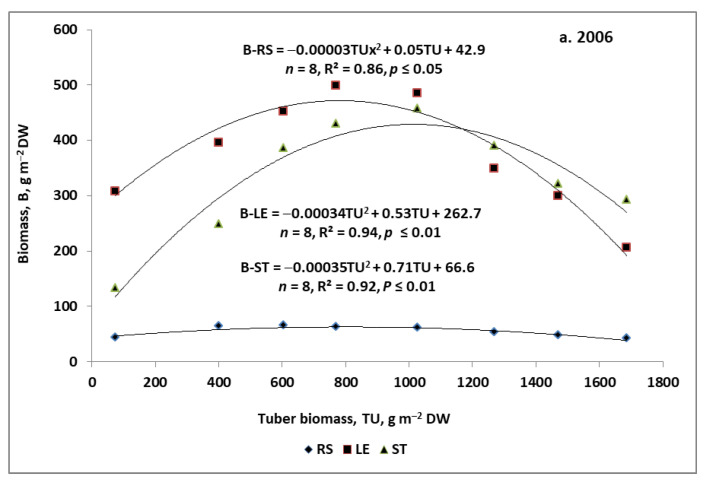

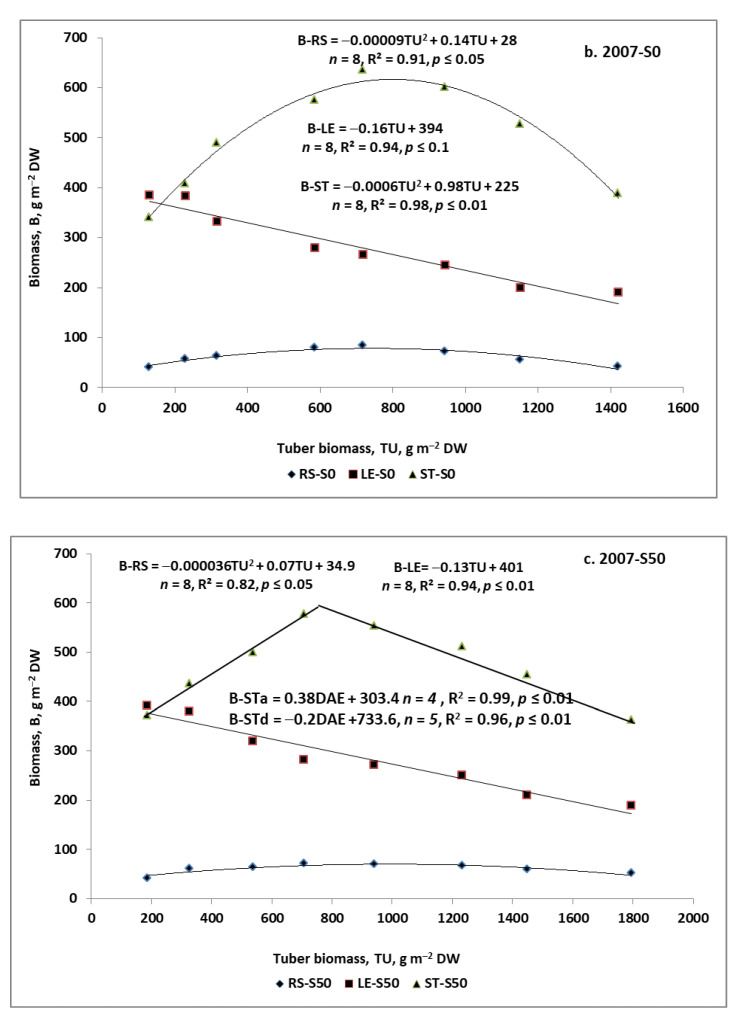
Sink–source relationships between the growth of tuber biomass and growth of competitive organs. (**a**) 2006; (**b**) 2007-S0; (**c**) 2007-S50. Legend: RS—roots and stolons, LE—leaves, ST—stems, TU—tubers; B-STa—ascending phase; B-STd—descending phase.

An analysis of the 2006 dataset clearly highlights that the stems and leaves were the most unstable part of the potato in response to the seasonal dynamics of tuber growth (Figure 5a). The stems showed the highest growth dynamics, i.e., a considerable increase in their own biomass per unit of tuber biomass in the ascending phase, and a mild decrease in the descending phase. The reverse pattern was noted for leaves.

For 2007, the CI indices are presented for the main treatments (Figure 5b,c):

1. S0:
  - Ascending phase: 0.07 (RS) < 0.48 (ST, g g^−1^ tuber DW);
  - Descending phase: −0.45 (ST) < −0.13 (LE) < −0.06 (RS g g^−1^ tuber DW).
2. S50:
  - Ascending phase: 0.07 (RS) < 0.48 (ST, g g^−1^ tuber DW);
  - Descending phase: −0.45 (ST) < −0.13 (LE) < −0.06 (RS g g^−1^ tuber DW).

In 2007, the most distinct seasonal trends in biomass development were observed for leaves. The stagnation phase was revealed in the early stages of tuber growth. In the case of the plot fertilized only with N (the S0 plot), this phase extended from the 35th to the 55th DAE. For the S50 variant a slightly shorter lag-phase was observed. In subsequent stages of potato growth, the leaf biomass showed a mild decline, lasting until harvest (Figure 5b). In the main sulfur-fertilized plot (S50), the descending LE phase began as early as on the 45th DAE, showing a mild decline towards maturity (Figure 5c). The stems showed markedly different growth trends in response to S application. In the S0 plot, the ST growth rate in both stages was both the highest and the most long-lasting. In the main plot with N + S addition, the ST growth rate in the ascending phase was only slightly lower than in the S0 variant, but the rate of decline in the descending phase was twice as low.

### 2.4. The In-Season Growth and Partitioning of Total Dry Matter

The seasonal growth trends in total potato biomass were variable in response to experimental factors. In 2006, significant and, at the same time, stable differences between fertilization treatments appeared at the earliest on the 75th DAE and persisted until harvest. Considerably higher potato biomass (B) was recorded in the N60S50 and N120S0 plots (Figure S1a). The trends obtained generally best fit the quadratic regression model. However, DAE_op_ during the growing season, as recorded on the 102nd DAE, was only achieved for the N60S0 plot. In the case of other treatments, DAE_op_ appeared after the harvest. Despite the seasonal variability of B, the tuber yield was independent from the experimental factors (Figure 1a). In 2006, the increase in B during the growing season, calculated on the basis of averaged data, was consistent with the quadratic regression model (Figure S2a):

(6) B = −0.245DAE^2^ + 55.5DAE − 867.6 for *n* = 10, R^2^ = 0.98 and *p* ≤ 0.01

The obtained trend clearly indicates that the theoretical increase in potato biomass in 2006 was longer than the date on which the potatoes were harvested. The optimal harvest date, as indicated by the DAE_op_, would be reached on the 113th DAE.

In 2007, the increase in B during the growing season was variable, but significantly driven by the S × T interaction (Figures S1b,c and S2b,c). Generally, the linear trend best describes the in-season increase in Bs:

(7) S0: B = 20.8 DAE + 37.4 for *n* = 19, R^2^ = 0.95, *p* ≤ 0.01

(8) S50: B = 24.1 DAE + 15.1 for *n* = 19, R^2^ = 0.97, *p* ≤ 0.01

The obtained models clearly show that the in-season growth of B on the sulfur-fertilized plot was 16% faster than on plots with N only. The differences, first observed on the 25th DAE, persisted until harvest. Significant differences between the two treatments did not become significant until the 55th DAE. Greater B in the plots treated with S was recorded in the final stages of potato yield development. As a result, the yield of tubers at harvest was 26% higher on the plots fertilized with sulfur compared to those fertilized only with N.

In both study years, regardless of the course of the weather, the value of the potato harvest index PHI increased progressively in the growing season (Table 1). The in-season trend of PHI, regardless of the year and fertilization treatments, was consistent with the linear regression model and was significant only for the observation (T):

(9) 2006: PHI = 0.8 DAE − 7.28 for *n* = 8, R^2^ = 0.95, *p* ≤ 0.01

(10) 2007: PHI = 0.8 DAE − 12.6 for *n* = 8, R^2^ = 0.99, *p* ≤ 0.01

The main difference between the two equations is the constant, which was much lower in 2007. The difference between the years began directly at DAE 35, reaching the highest values in the tuber bulking phase (from 45 to 55 DAE). In 2006, PHI in this particular period was more than 10% higher than in 2007. Final PHI in 2006 reached 76% and in 2007 it was 72% (Figure S3).

**Figure 6:**
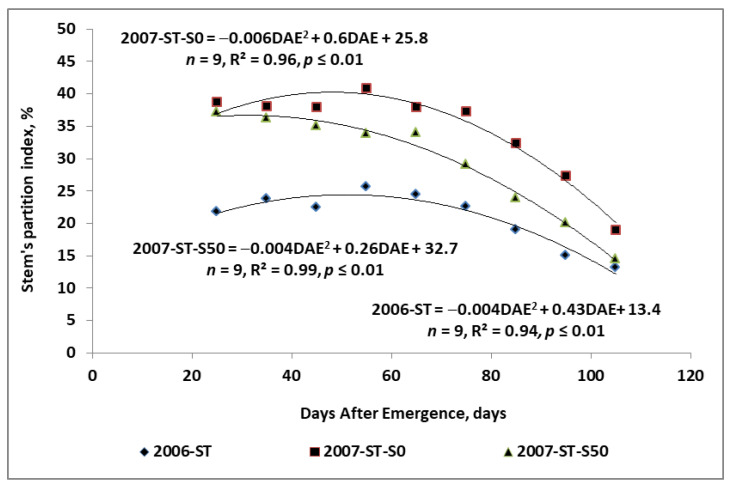
Seasonal trend in the relative share of stem biomass in the total potato biomass. Legend: N60, N120—nitrogen rates of 60 and 120 kg ha^−1^; S0, S50—sulfur rates of 0 and 50 kg ha^−1^.

The seasonal structure of the dry matter partition between potato organs is shown in Figure S3. For each part of the potato competing with tubers, a seasonal downward trend in the share of biomass during the growing season was observed. The relative share of roots + stolons in the B decreased according to the power function in 2006, and linearly in 2007. The seasonal trend in leaf biomass in the B, regardless of the year and fertilization treatments, corresponded best to the power function. Much more diverse patterns were observed for the stem biomass, which was best described by the quadratic regression model (Figure 6). The differences between the years refer to:

(1) Share of the stem biomass in the B in the early stages of tuber growth;
(2) The length of the stability period of stem biomass share.

In 2006, the percentage of stems in total potato biomass ranged from 22% on DAE 25 to a maximum of 26% on DAE 55. At harvest, it declined to 13%. In 2007, the initial share of stems in DAE 25 was 38%. The application of sulfur significantly influenced the general trend of stems contribution in the total potato biomass. Their share on the S0 plot showed a significant increase, reaching 41% on the 55th DAE, while on the S50 plot it decreased smoothly from DAE 35. The stabilization phase, assuming a 4% difference between consecutive observations was 50 days in 2006, while in 2007 it reached 60 days for the S0, and 50 days for the S50.

## 3. Discussion

### 3.1. Tuber Yields and Seasonal Trends of Potato Tubers Growth

The in-season development of the potato tuber yield is a function of the weather conditions during the growing season and the supply of nutrients [12,14]. Our study confirmed this opinion, but only partially. In this particular case, the final yield of tubers, however, was not dependent on the course of the weather, even though the weather conditions were completely different. Under climatological conditions in Poland, the highest tuber yield can be obtained provided there is medium precipitation in May (45 mm) and June (65 mm), and high precipitation in July (90 mm) and August (75 mm) [23]. The environmental conditions, evaluated based on the Sielianinov hydrothermal indices, indicated a severe drought in 2006 (0.58), and optimal weather conditions in 2007 (1.34) [24]. The final tuber biomass averaged for experimental treatments, was 1687 g m^−2^ DW in 2006 and 1608 g m^−2^ DW in 2007. The biomass of tubers recalculated into fresh weight was 84.3 t ha^−1^ (20% DM content) in 2006, and 86.6 t ha^−1^ (18.5% DM content) in 2007. Tuber yields at this level under natural precipitation are possible, but only if the soil is very fertile [25]. In the dry 2006, regardless of the N rate, which was 60 and 120 kg ha^−1^, the tuber yield was at the same level. The lack of response by the potatoes to N doses indicates a high mineralization potential of the soil to supply the plants with N in the phases of intensive tuber growth [3,26]. A positive impact of S^0^ on tuber biomass was found each year, but it was significant only in the optimal 2007 (Figure 1b). At harvest, the average biomass of potato tubers on the main plot fertilized only with N was lower by 21% than that receiving sulfur at the rate of 50 kg ha^−1^. The significant increase in the tuber yield due to S fertilizer can be partly explained by the content of its available form in the soil prior to planting the potatoes. The data obtained support the results of other studies, but the effect of S in our study was more striking [27,28]. On the basis of the tuber yield, it can be stated that S is an effective yield-forming factor for potatoes.

The seasonal pattern of potato tuber yield growth, confirming the physiological maturity of tubers, should meet the conditions of the quadratic or any sigmoidal regression model [14]. In our study, the seasonal trends in tuber biomass growth were only seemingly consistent with the quadratic regression model in 2006, and the linear regression model in 2007. In 2006, the DAE_op_ for B appeared shortly after the harvest date, indirectly indicating the slightly immature status of harvested tubers. The linear model obtained in 2007 is a direct indicator of the immature physiological status of potato plants at harvest [29].

The general, linear trend of tuber yield increase during the growing season is consistent with the theory, indicating a dependence of the crop yield on the source–sink relationship during the growing season [30,31]. On the basis of the linear growth pattern of potato biomass, it can be concluded that under favorable environmental and soil conditions for the potato, tuber yield is determined by the sink strength. This means that tuber activity in the studied case was the yield driver that affected the source activity. This type of seasonal tuber yield growth suggests, however, that the yielding potential of the tubers, i.e., their sink capacity for assimilates, was not realized in the studied case [14].

### 3.2. Growth Competition between the Tuber and Non-Storage Potato Organs

In the classical approach to the source–sink relationship, the strength of the physiological sink depends on the source activity [2]. This hypothesis was verified during this study. The yield of potato tubers can be evaluated as a result of the relationships between the seasonal growth rate of tubers and the growth rate of other potato organs, which compete with tubers for assimilates. The set of competitive potato organs includes leaves, steams, stolons, and true roots [7]. The obtained linear model of tuber growth during the growing season, regardless of weather conditions, clearly indicates that the enlarging tubers act as a pure physiological sink (Figure 1).

The source–sink relationships in potatoes require recognition of the state of its organs’ biomass, in response to tuber initiation. As it results from the analysis of the dynamics of the growth of potato organs, the impact of tuber initiation on the source growth depended on the course of the weather during the pre-tuberization stages of potato growth. The growth habit of vines in response to weather in both years was different. The pattern of leaf growth was governed up to early tuber bulking by temperature. In 2006, leaves stopped growing on the 66th DAE, whereas in 2007, it was 18 days earlier (48th DAE). The main reason for the observed differences was the temperatures in July. In 2006, air temperature exceeded 25 °C, but in 2007, it was below 20 °C. Under relatively low temperature the numbers of branches and leaves associated with them are lower. The change point is 25 °C, which results in the appearance of long stems, but with smaller leaves [32]. The tuber sink strength, defined by its initial weight at the beginning of the bulking phase, determines its requirements for assimilates in the subsequent stages of growth [13]. In potatoes, leaves are a pure physiological source of assimilates, which also act as a temporary sink, using a part of the fixed CO_2_ to build their own biomass [7,33]. Other vegetative organs that compete with the enlarging tubers can also be regarded as temporary sinks. The critical value of the temporary tuber biomass (TTB_crit_) was used as a criterion for evaluating the in-season competition between enlarging tubers and the growth rate of other potato organs. TTB_crit_ defines the critical growth rate of the TTB required to break down the dominance of the potato organs that compete with tubers for assimilates. The TTB_crit_ cannot be treated as a single point. It can be considered as a period of a different length, in which the growth rate of tubers and the competitive organ are temporarily balanced. Based on TTB_crit_, the entire growth period of potato vegetative parts competing with the growing tubers can be divided into three sub-phases:

(1) Ascending, having the status of a temporary sink;
(2) Stagnating, which is a transition stage between the sink and a pure source phase;
(3) Descending, having the status of a pure source.

The property of the first period is a net gain in the biomass of all potato organs. This state indicates a lack of competition between the potato organs for assimilates. The descending growth phase of potato organs competing with tubers is the period of pure remobilization of dry matter from each potato organ into enlarging tubers.

In both years, the growth of potato organs competitive with tubers did not change the linear pattern of tuber yield increments during the growing season (Figure 1). The tuber sink strength can therefore be considered as the factor forcing both the size of the pure source, i.e., leaf biomass, and the biomass of other temporary sink organs. These facts absolutely support the hypothesis by Körner [30,31], suggesting the primacy of the net sink over the net source.

The observed relationships cannot be explained without taking into account the course of weather and experimental factors. As reported by Li et al. [3], the in-season C reallocation is to some extent, modified by environmental and agronomic factors. In our case, in the dry 2006, the relationship between the biomass of tubers and leaves during the growing season was described by the quadratic regression model. In 2007, the seasonal trend in leaf biomass showed a completely different pattern. As a rule, leaf biomass decreased from the beginning of tuber enlargement. The degree of the LE decline, supported by values of the direction coefficient, was steeper on the plot fertilized only with N. This type of LE trend can be explained by the accelerated rate of leaf dry matter remobilization due to the progressively increasing pressure of the enlarging tubers [14]. Potato plants fertilized with S showed a much slower depression of LE biomass in the descending phase of this organ development. As a consequence, the final tuber yield on plots fertilized with both N and S was 26% higher. The obtained final result of the tuber yield not only confirms, but also explains the reason for the high response of the potato to the application of sulfur [27,28].

The production functions of leaves in potatoes can be explained by analyzing the seasonal trends of LE. They are described by indicators such as DAE_crit_ and DAE_op_. It should be strongly emphasized that the DAE_crit_ for LE in 2006 appeared long before the DAE_op_, but in 2007 it was much later. In this particular year, the difference between both indices, expressed as DAE_diff_, was negative. The difference obtained clearly indicates that tuber biomass exceeded that of leaf biomass before it reached its maximum. Thus, in full potato vegetation, DAE_crit_ for LE can be treated an important indicator of the final tuber yield:

(11) TBU = −63.8D_crit_ + 4728 for *n* = 3, R^2^ = 0.98, and *p* ≤ 0.01

The equation obtained confirms the occurrence of competition for assimilates between leaves and progressively growing tubers. This type of relationship between the growth of leaves and tubers stresses the often-discussed fact of the excessive growth of leaves as a factor disturbing the growth of tubers [33]. In our study, the observed competition revealed itself under a certain set of environmental and agronomic conditions, i.e.:

(1) In a year of favorable growth conditions for potatoes at the beginning of vegetation;
(2) On soils with high natural fertility (high content of humus and available nutrients);
(3) Under a good supply of nitrogen, including its applied dose.

In agronomic practice, these conditions are frequently observed under irrigation [34]. Our study showed that the application of 50 kg S ha^−1^ in the form of elemental sulfur resulted in significant DAE_crit_ shortening. As a result, the final tuber yield in the N + S variant approached the maximum value in the studied case.

The missing information in tuber yield development with respect to the in-season competition between tubers and non-storage potato organs mainly refers to the production function of the stems. This potato tissue, as a temporary sink, is rich in proteins, carbohydrates, and mineral nutrients [32,35]. The growth rate of the stem biomass in the ascending phase was far below 1.0, which only seemingly stresses the lack of competition for assimilates with tubers. In both years, the stem growth rate in the ascending phase was sufficiently high, but did not exceed +0.45 g g^−1^ of tuber DW. In the descending phase, it was significantly affected by year and fertilization treatments. In 2006 for all treatments and in 2007 for treatments with S, this parameter was almost similar, varying from −0.26 to −0.23 g g^−1^ tuber DW. In 2007, this parameter on plots fertilized only with N was −0.45 g g^−1^ of tuber DW. A much lower rate of stem biomass decrease on plots fertilized with S, concomitant with a higher tuber yield, suggests that the stems act as an assimilate buffer for the growing tubers. The quadratic regression model of the relationship between tubers and stems growth, dominating in all treatments, indicates the presence of the stagnation period in the main phase of tuber bulking, which stabilized tuber growth (Figure 5). The buffer function of stems was also corroborated by their share in the total tuber biomass (Figure 6). The rate of stem growth was significantly modified by application of elemental S. The observed response of the stem biomass to environmental factors and agronomic measures corroborates the hypothesis of stem plasticity to the growing conditions [36]. This specific function of the potato stem largely explains the increase in tuber yield in response to S application [25,26].

White et al. [6] concluded that the increase in crop productivity requires a quantitative definition of the extent to which sources or sinks limit crop plant growth, and this changes during development. The quantitative relationships between the potato organs during the growing season are much more complex than is often assumed in even very sophisticated models of potato growth and yielding [3,8]. Our study provided not only a few sets of basic data on seasonal trends in the biomass of roots + stolons, leaves, stems, and finally tubers, but also showed allometric relationships between tubers and non-storage potato organs.

## 4. Materials and Methods

### 4.1. Experimental Site

A field experiment was carried out at Kicin (52°46’ N, 17°02’ E, Poland) on soil originating from a silty clay loam classified as Chernozems loamic [36]. The content of organic (C_org_) in a 0.0–0.3 m layer was 33 ± 0.9 and 27 ± 0.1 g kg^−1^ soil (loss-on ignition); pH was 6.6 and 7.0 (1.0 M KCl) in 2006 and 2007, respectively. The content of available nutrients, measured before the application of fertilizers, was very high for P (305 ± 71 and 267 ± 28); medium and low for K (253 ± 117 and 160 ± 13); low for Mg (117 ± 14 and 106 ± 39); low for Ca (1605 ± 137 and 1678 ± 618) mg kg^−1^ soil for 2006 and 2007, respectively (Mehlich 3 method) [37,38]. The amount of the mineral N (N_min_), measured in a 0.0–0.6 m layer, was 60 ± 6 and 83 ± 11kg ha^−1^ in 2006 and 2007, respectively [39]. The amount of available sulfur (S-SO_4_) was 10.3 ± 2.4 and 7.4 ± 1.0 mg kg^−1^ dry soil, in 2006 and 2007, respectively [40].

### 4.2. Weather Conditions

The in-season differences in potato biomass resulted from the course of the weather in the early stages of plant growth. In both years, the average monthly temperatures were higher than the long-term averages (1965–2007; 14.6 °C in May, +0.6 °C and +2.0 °C; June, 17.7 °C, +3.3 °C and +3.7 °C, in 2006 and 2007, respectively). July 2006 (20.1 °C) was extremely hot (+5.6 °C), and in 2007 it was much cooler (−0.5 °C). The total amount of precipitation in 2006 and 2007 for these three months amounted to 108 mm and 233 mm, respectively. The growth conditions evaluated on the basis of Sielianinov hydrothermal indices were significantly worse in 2006 (0.58, severe drought), compared to 2007 (1.34, optimal) [29]. The shortage of precipitation in 2006, due to the high content of soil water and nutrient resources at the time of potato planting did not disturb the development of potato biomass. The growth conditions in 2006 were significantly improved due to extremely high precipitation in August (150 mm).

### 4.3. Treatments and Crop Management

The field experiment was arranged as a two-factorial split-plot design, replicated 8-folds:

1. N rate (acronym N): 60 and 120 kg N ha^−1^;
2. Sulfur: without S (S0), with sulfur (S50);
3. Periodic sampling of potato plants during the potato growing season was used as a third experimental factor [40].

Nitrogen in the form of urea was applied in accordance with the experimental design in the whole rate prior to potato planting. Phosphorus at a rate of 25.8 kg P ha^−1^ as triple superphosphate (46% P_2_O_5_); K at a rate of 99.6 kg K ha^−1^ as muriate of potash (KCl); and sulfur as elemental sulfur (S^0^) at a rate of 0, and 50 kg ha^−1^ were applied together with N two weeks before potato planting. The total area of a single plot was 58.5 m^2^ (13 × 6 m) The potato variety *Zeus* was planted on the 24 April 2006 and 19 April 2007, respectively. The preceding crop to potato was spring barley. The post-barley agronomic operations for potato planting in the spring of the following year were aimed at reducing water losses and weed growth. To achieve this goal, the soil was harrowed several times after plowing. A total of 53,000 potato tubers were planted in 0.75 m rows and at 0.25 m distance within a row. Plant protection was carried out in accordance with the code of good practice. The tubers were harvested mechanically 105 days after emergence from an area of 19.5 m^2^ (13 × 1.5 m).

### 4.4. Plant Material Sampling and Analysis

The plant material used for dry matter determination was collected from 8 plants (1.0 × 1.5 m = 1.5 m^2^) per plot. Periodic sampling was performed at 10-day intervals, starting on the 15th day after full emergence until maturity (Days After Emergence, DAE): 15, 25, 35, 45, 55, 65, 75, 85, 95, and 105. Samples were taken from the soil to a depth of 30 cm from the top of the ridge. The sampled material was then divided, depending on the potato stage of growth, into subsamples of leaves (LE), above-ground stems (ST), stolon + roots (RS), and tubers (TU). The results are expressed on a dry weight basis.

### 4.5. Calculated Parameters

#### 4.5.1. Growth Pattern of Potato Organs

The general pattern of the in-season potato biomass development (RS, LE, ST, total biomass—B, temporary tuber biomass—TTB) was obtained after fitting the actual results to polynomial regression models:

(12) Linear: TTB=aDAE+B

(13) $$\mathrm{Quadratic}:\;\mathrm{TTB}={\mathrm{aDAE}}^{2}+\mathrm{bDAE}+C$$

The main estimated parameters of this function are:

(14) $${\mathrm{DAE}}_{\mathrm{op}}=-\frac{b}{2a}$$

(15) $${\mathrm{TTB}}_{\max}=\;c-\frac{b^{2}}{4a}$$

where: TTB—temporary tuber biomass, g m^−2^ DW; B_max_—maximum biomass of a particular potato organ, g m^−2^ DW; DAE—Days After Emergence; DAE_op_—the optimum DAE; a, b, c, d—regression constants.

#### 4.5.2. Critical Day of Tubers Growth—DAE_critt_

Critical day of tuber growth was calculated by solving three pairs of corresponding equations, describing the seasonal growth trends of the biomass of potato organs:

TU vs. RS, b. TU vs. LE, c. TU vs. ST.

#### 4.5.3. Competition Indices

Competition indices were calculated on the basis of the appropriate pair of linear equations, describing the ascending and descending trend in biomass growth of potato organs competing with tubers. Based on the obtained linear models, the following set of parameters was calculated for each potato organ:

1. CI index—the competition index, i.e., the growth rate of a specific potato organ per unit of tuber biomass. This parameter is equal to the value of the slope of the obtained linear equation (CD), g g^−1^ tuber;
2. TTB_opt_—optimal value of the temporary tuber biomass for the maximum biomass of the competing potato organ, g m^−2^ DW;
3. B_max_—maximum biomass of the potato organ competing with the tubers, g m^−2^ DW.

The general concept of the analysis of competitive relations was divided into two steps:

- Ascending sub-phase: B_as_ = a_as_TU + b_as_;
- Descending sub-phase: B_d_ = −a_ds_TU + b_ds_.

### 4.6. Data Analysis

The collected data were subjected to an analysis of variance using STATISTICA^®^ 13 (StatSoft, Inc., Krakow, Poland 2013). Experimental factors, i.e., N and S rates, including observations (T) were treated as fixed effects [41]. The influence of the year was analyzed independently in order to assess the seasonal variability of examined potato traits on the experimental factors. The distribution of the data (normality) was checked using the Shapiro–Wilk test. The homogeneity of variance was checked by the Bartlett test. Means were separated by honest significant difference (HSD) using Tukey’s method, when the *F*-test indicated significant factorial effects at the level of *p* < 0.05. Polynomial effects were used to determine (i) linear and/or quadratic growth trends of potato organ biomass, (ii) growth rates of potato organs during the season.

## 5. Conclusions

The potato growth pattern coded at the onset of tuberization was a decisive factor for the dry matter partitioning between the potato organs during the subsequent tuber growth phase. The tuber sink strength, expressed as a linear increment of tuber biomass in the growing season, was a key driver influencing the seasonal growth patterns of potato organs, such as leaves, stems, and roots and stolons, which competed with tubers for assimilates. Two indicators have been developed to explain the relationship between growing tubers and potato organs competing for assimilates during the growing season. The first, DAE_crit_, defined as DAE, at which the tuber biomass exceeds that of the competing potato organ. It can be concluded that under particular growing conditions, when DAE_crit_ precedes the maximum leaf biomass, the tuber yield decreases. This phenomenon occurred when the potatoes were fertilized only with N. Sulfur fertilization was a remedy for the negative effects of nitrogen applied alone. The second index, the competition index (CI), has proven to be a reliable indicator of the seasonal evaluation of the tuber growth rate and competitive potato organs. Its prognostics value was especially revealed in the descending phase of stem growth. It can be concluded that the absolute rate of stem biomass decrease above ¼ of the tuber growth rate reduces the tuber yield.

The tuber yield-forming effect of added sulfur results from a balanced growth of stems during the ascending and the descending phase. The stabilizing action of sulfur resulted in a smooth change of the stem biomass during the descending phase. As a consequence, there was a significant reduction in the value of the CI compared to the effect of nitrogen applied alone (−0.23 g g^−1^ DW vs. −0.45 g g^−1^ DW for the S50 to S0 plots, respectively). Lower values of this index, both in the ascending and descending phase of the seasonal growth rate of stem biomass, resulted in a higher yield of tubers. Potato stems can be, therefore, treated as a tuber yield stabilizer, buffering the transfer of assimilates from leaves to the enlarging tubers.

This study clearly showed that allometric relationships between tubers and non-storage potato organs growth during the potato growing season are key factors determining the final tuber yield. The use of elemental S significantly modifies the yield-forming effect of nitrogen, leading to a higher yield of tubers.

## Figures and Tables

**Table 1:** Means of response variables and significance of F values for fixed sources of variation during the potato growth season.

Source of Variation	Factor Level	Degree of Freedom	Roots and Stolons	Leaves	Stems	Tubers	Total Biomass	Tuber’s Harvest Index	Roots and Stolons	Leaves	Stems	Tubers	Total Biomass	Tuber’s Harvest Index
			g m^−2^ DW					%	g m^−2^ DW					%
			2006						2007					
Nitrogen (N) kg ha^−1^	60	1	48.2	334.6	304.9	910.9	1385.9	48.6	51.7	245.8	414.0	861.6	1359.4	47.2
	120		49.3	312.7	298.3	915.5	1361.5	49.1	56.3	282.4	490.1	721.0	1356.6	39.2
F value and significance			2.3 ^ns^	6.8 **	0.9 ^ns^	0.1 ^ns^	2.1 ^ns^	0.7 ^ns^	29.3 ***	43.8 ***	29.3 ***	43.8 ***	68.3 ***	94.9 ***
Sulfur (S) kg ha^−1^	0	1	48.4	333.3	302.6	897.6	1371.6	48.3	54.7	263.3	463.7	686.8	1285.3	39.8
	50		49.3	314.0	300.6	914.5	1375.8	49.4	53.4	265.4	440.3	896.0	1430.7	46.6
F value and significance			0.84 ^ns ‡^	5.3 *	0.1 ^ns^	6.8 **	0.06 ^ns^	4.5 *	2.3 ^ns^	0.1 ^ns^	2.3 ^ns ‡^	0.1 ^ns^	6.4 **	65.6 ***
Sampling time (T), DAE ^1^	15	9 ^x^ 8 ^y^ 7 ^z^	17.9	51.2	nd ^†^	nd	69.1	nd	7.7	71.4	nd ^†^	nd	79.1	nd
	25		36.1	187.4	61.9	nd	285.4	nd	49.5	282.9	210.3	nd	542.6	nd
	35		42.6	308.6	131.9	73.4	556.5	16.0	40.2	388.1	355.6	158.0	941.9	13.2
	45		63.0	396.5	247.1	400.5	1107.0	23.0	58.1	381.6	421.4	277.4	1138.5	35.9
	55		64.9	452.6	384.7	604.9	1507.0	30.8	62.7	325.5	493.6	426.1	1307.9	41.0
	65		61.5	498.6	429.7	770.2	1759.9	40.0	75.0	280.6	575.6	645.9	1577.1	43.8
	75		60.3	485.5	456.5	1027.3	2029.6	46.0	76.6	268.6	593.3	828.4	1766.9	50.4
	85		52.2	349.8	389.5	1269.6	2061.0	54.9	68.3	247.8	555.0	1087.5	1958.6	61.9
	95		47.3	300.4	320.9	1469.0	2134,5	62.9	56.3	204.9	489.3	1299.3	2049.8	69.0
	105		41.9	206.1	292.2	1686.7	2226.9	72.0	46.0	189.8	374.2	1608.0	2218.1	75.8
F value and significance			160.0 ***	117.5 ***	162.3 ***	1136.1 ***	930.0 ***	671.2 ***	222.6 ***	116.3 ***	222.6 ***	116.3 ***	81.0 ***	277.7 ***
Significance of interactions ^‡^														
N × S		1	*	***	***	^ns^	***	***	^ns^	^ns^	^ns^	***	***	***
N × T		9 ^x^,8 ^y^,7 ^z^	***	^ns^	***	*	**	*	^ns^	***	**	^ns^	^ns^	^ns^
S × T		9,8,7	***	^ns^	^ns^	^ns^	^ns^	^ns^	***	*	**	**	**	ns
^N^ ^× S^ ^× T^		^9,8,7^	^***^	^***^	^***^	^ns^	^***^	^***^	^**^	^ns^	^ns^	^ns^	^ns^	^ns^

^‡ ns =^ Non-significant at *p* ≤ 0.05; *, **, ***; significant at *p* ≤ 0.05, 0.01, 0.001,. ^†^ Non-determined; ^x^ denotes roots + stolons, leaves, total biomass; ^y^ stems; ^z^ tuber dry weight and; ^z^ also tuber harvest index. Within a column, means followed by same lowercase letter are not significantly different at *p* ≤ 0.05. Letters are not shown for significant main effects that also had a significant interaction effect. ^‡^ Means comparison for variables having a significant interaction effect are shown in Figure 1, Figure 2, Figure 3 and Figure 4 or discussed in text. Legend: DAE ^1^—Days after Emergence.

**Table 2:** Indices describing competition between growth of tubers and non-storage organs.

Plant Part	DAE_op_	DAE_crit_	DAE_diff_	B_max_, g m^−2^ DW
2006				
Roots + stolons, RS	66.1	33.0	33.1	63.4
Leaves, LE	64.7	47.2	17.5	470.4
Stems, ST	66.4	37.4	29.0	422.2
2007, S0				
Roots + stolons, RS	67.9	31.8	36.1	74.5
Leaves, LE	41.7	51.9	−10.2	403.3
Stems, ST	73.7	58.6	15.1	590.4
2007, S50				
Roots + stolons, RS	70.5	27.0	43.5	70.0
Leaves, LE	46.0	46.4	−0.4	394.5
Stems, ST	70.3	51.5	18.8	551.2

Legend: DAE_op_—the optimum day for the maximum biomass of potato organs (B_max_) competing with tubers; DAE_crit_—DAE at which biomass of tubers exceeded biomass of a particular non-storage potato organ; DAE_diff_ = DAE_op_ − DAE_crit_.

**Table 3:** Indices describing competition between growth of tubers and competitive organs.

Ascending Phase	Descending Phase	TTB_crit_, g m^−2^ DW	B_max_, g m^−2^ DW
2006			
B-RS = 0.044 TUY + 41.1 *n* = 3, R^2^ = 0.90, *p* ≤ 0.05	B-RS = −0.03 TUY + 88.1 *n* = 4, R^2^ = 0.99, *p* ≤ 0.01	652.8	69.8
B-LE = 0.27 TUY + 288.1 *n* = 4, R^2^ = 0.99, *p* ≤ 0.01	B-LE = −0.041 TUY + 894 *n* = 4, R^2^ = 0.98, *p* ≤ 0.01	888.4	529.7
B-ST = 0.45 TUY + 91.9 *n* = 4, R^2^ = 0.90, *p* ≤ 0.05	B-ST = −0.26 TUY + 716.4 *n* = 4, R^2^ = 0.97, *p* ≤ 0.01	883.3	486.7
2007, S0			
B-RS = 0.067 TUY + 36.7 *n* = 5, R^2^ = 0.94, *p* ≤ 0.01	B-RS = −0.06 TUY + 127.6 *n* = 3, R^2^ = 0.99, *p* ≤ 0.01	693.9	85.3
Stagnation, (DAE 35 + 45)	B-LE = −0.13 TUY + 362.3 *n* = 6, R^2^ = 0.96, *p* ≤ 0.01	^-^	383.2
B-ST = 0.48 TUY + 300.7 *n* = 5, R^2^ = 0.96, *p* ≤ 0.01	B-ST = −0.45 TUY + 1029.2 *n* = 3, R^2^ = 0.99, *p* ≤ 0.01	788.4	676.0
207, S50			
B-RS = 0.06 TUY + 29.9 *n* = 4, R^2^ = 0.86, *p* ≤ 0.05	B-RS = −0.023 TUY + 91.4 *n* = 4, R^2^ = 0.97, *p* ≤ 0.01	741.0	74.4
Stagnation, (DAE 35 + 45)	B-LE = −0.13 TUY + 400.6 *n* = 8, R^2^ = 0.94, *p* ≤ 0.01	^-^	383.2
B-ST = 0.38 TUY + 303.4 *n* = 4, R^2^ = 0.99, *p* ≤ 0.01	B-ST = −0.23 TUY + 776.4 *n* = 4, R^2^ = 0.98, *p* ≤ 0.01	778.0	599.8

Legend: B—biomass, RS—roots and stolons, LE—leaves, ST—stems, TU—tubers, TUY—tuber yield; TTB_crit_—critical temporary tuber biomass, B_max_—maximum biomass of the competing potato part.

## Data Availability

The data presented in this study are available on request from the corresponding author. The data are not publicly available due to the private property of Karolina Frąckowiak.

## Supplementary Materials

The following are available online at https://www.mdpi.com/article/10.3390/plants11030248/s1, Trends in the biomass of potato organs increase during the growing season, Figure S1. Seasonal trends of potato biomass growth. (a) 2006; (b) 2007, Figure S2. Trends in the potato biomass increase and partitioning between organs, Figure S3. Seasonal variability in the relative share of potato organs in the total biomass.
