# Supplementary material for: Does Elemental Sulfur Act as an Effective Measure to Control the Seasonal Growth Dynamics of Potato Tubers (Solanum tuberosum L.)?
Source: Plants (Basel). 2022 Jan 18;11(3):248. doi: 10.3390/plants11030248 (PMC8839987; doi:10.3390/plants11030248)
Supplement: Supplementary file 1 [file plants-11-00248-s001.zip › plants-1506600-supplementary.pdf]

Supplementary Materials

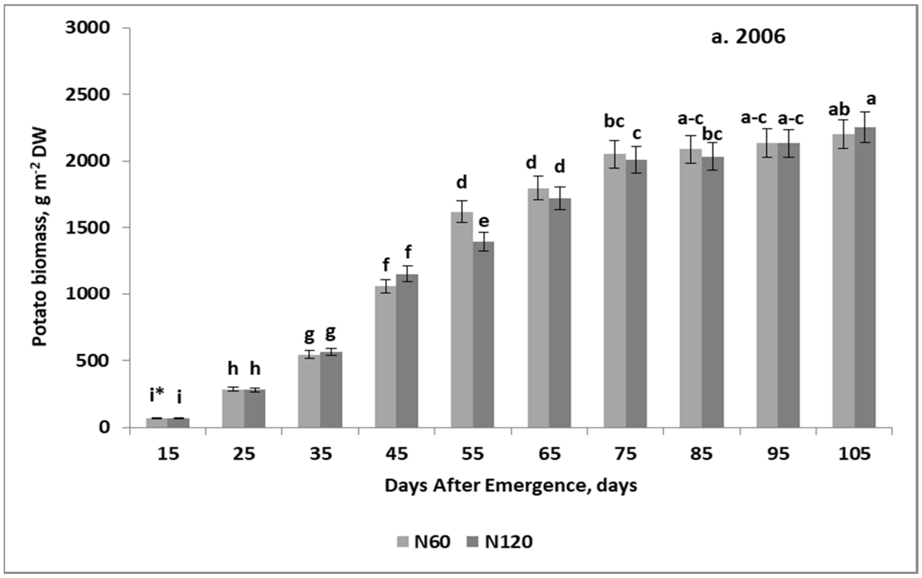

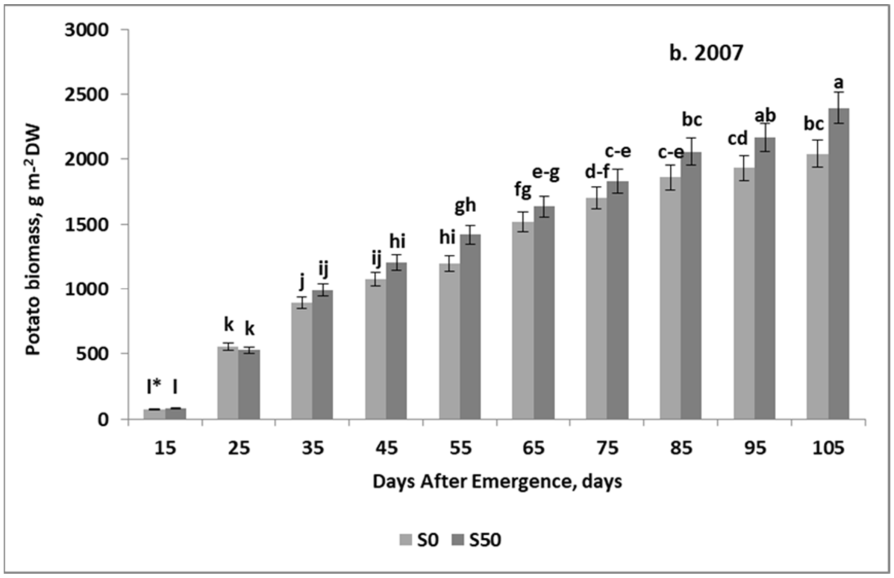

Figure S1. Seasonal trends of potato biomass growth. (**a**) 2006; (**b**) 2007. * HSD—calculated separately for each year; letters indicate significant differences between treatments ($p < 0.05$). Legend: S0, S50—sulfur rates of 0 and 50 kg ha^{-1}. Vertical bars represent standard error.

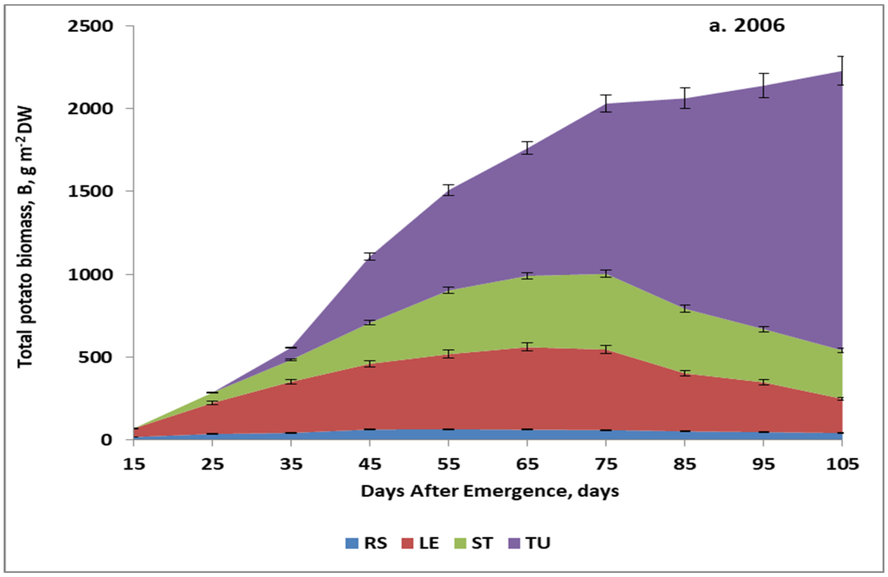
a. 2006
Total potato biomass, B, g m-2 DW
2500
2000
1500
1000
500
0
15
25
35
45
55
65
75
85
95
105
Days After Emergence, days
RS
LE
ST
TU

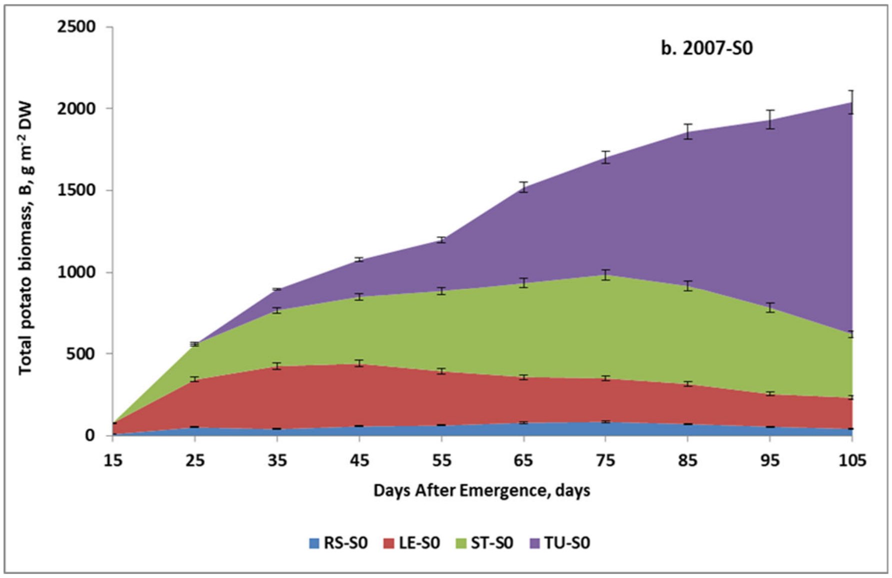
b. 2007-S0
Total potato biomass, B, g m-2 DW
2500
2000
1500
1000
500
0
15
25
35
45
55
65
75
85
95
105
Days After Emergence, days
RS-S0
LE-S0
ST-S0
TU-S0

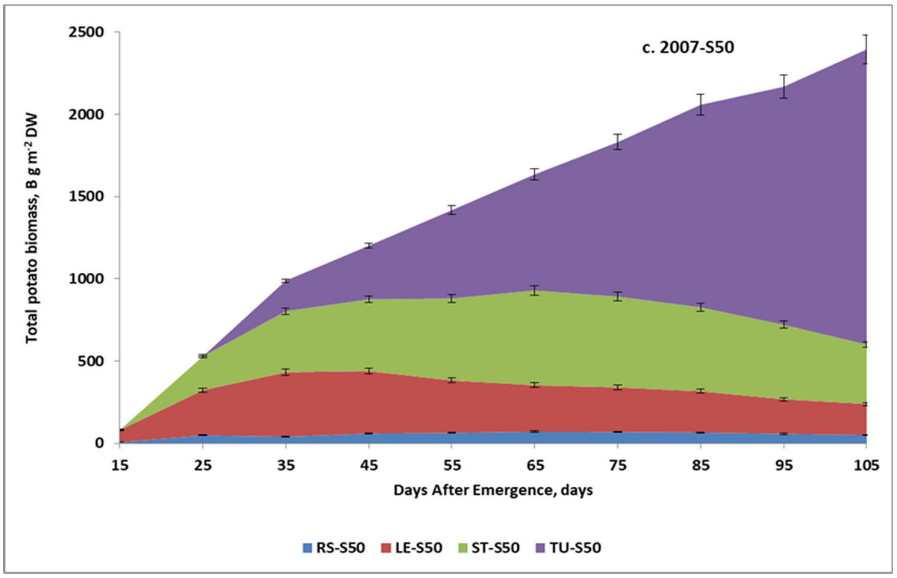

Figure S2. Trends in the potato biomass increase and partitioning between organs. (**a**) 2006; (**b**) 2007-S0; (**c**) 2007-S50. Legend: N60, N120—nitrogen rates of 60 and 120 kg ha^{-1}; S0, S50—sulfur rates of 0 and 50 kg ha^{-1}. Vertical bars represent standard error.

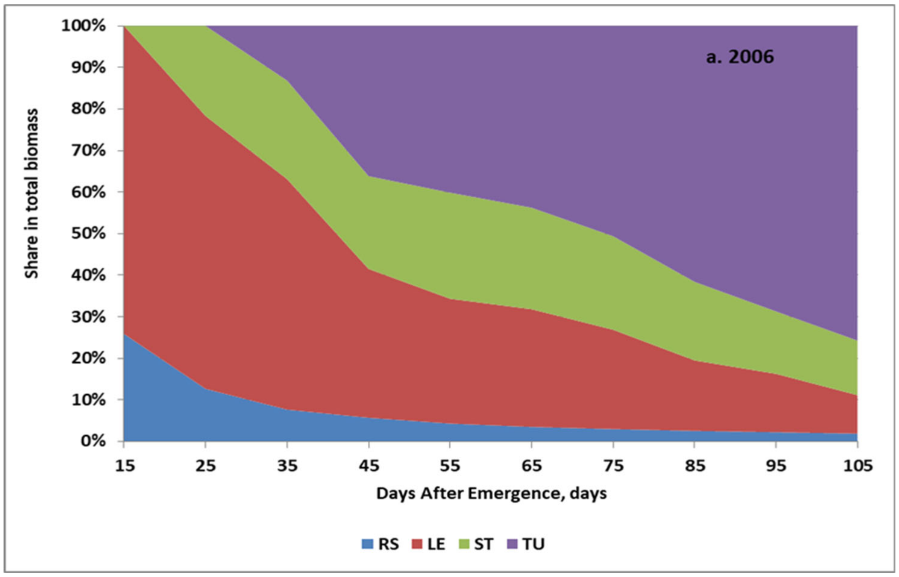

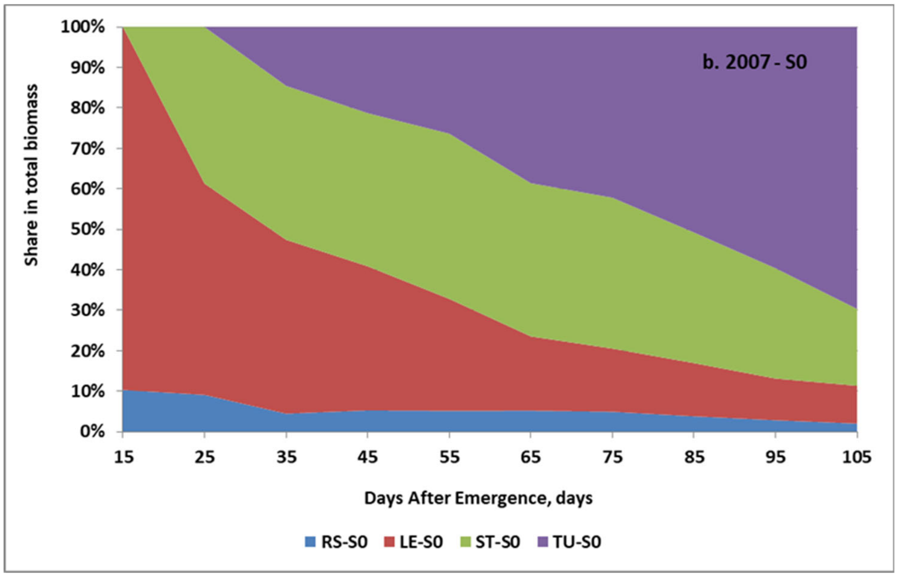

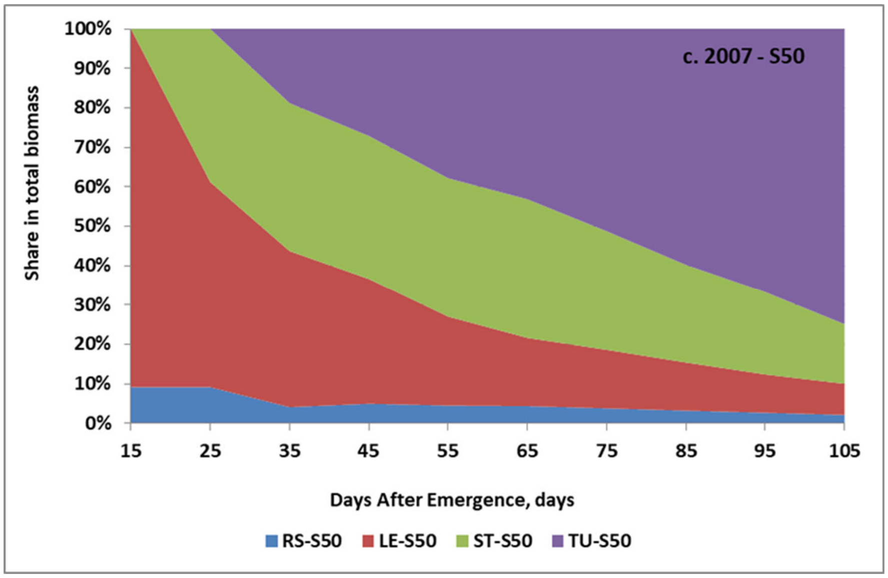

Figure S3. Seasonal variability in the relative share of potato organs in the total biomass. (**a**) 2006; (**b**) 2007-S0; (**c**) 2007-S50. Legend: RS—roots and stolons, LE—leaves, ST—stems, TU—tubers; S0, 50—sulfur rates of 0.50 kg ha^{-1}.
